# Primary Headache Approach in the Emergency Departments: A Systematic Scoping Review of Prospective Studies

**DOI:** 10.7759/cureus.36131

**Published:** 2023-03-14

**Authors:** Carlos M Ardila, Daniel Gonzalez-Arroyave, Santiago Angel, Mateo Zuluaga-Gomez

**Affiliations:** 1 Epidemiology and Public Health, University of Antioquia, Medellin, COL; 2 Surgery, Hospital San Vicente, Medellin, COL; 3 Neurology Department, Universidad de Antioquia, Medellín, COL; 4 Emergency Department, Hospital San Vicente Fundacion, Medellin, COL

**Keywords:** systematic scoping review, neuroimaging, red flags, emergency department, primary headache

## Abstract

This systematic scoping review aims to answer questions related to the main characteristics of primary headache, the need for neuroimaging, and the presence of red flags in these patients. A review of prospective studies including the MEDLINE/PubMed, Scopus, LILACS, and SCIELO databases, as well as the grey literature, was conducted. The methodological quality of the selected investigations was also assessed. Six investigations met the selection criteria. The mean age of people with primary headache was less than 43 years, with ages ranging from 39 to 46 years. Most of the studies reported the presence of nausea/vomiting, between 12% and 60% of the patients studied. To a lesser extent, there was also the presence of intense and moderate pain, loss of consciousness, stiff neck, presence of aura, and photophobia. The most frequent diagnoses were unspecified headache, migraine, and tension headache. The studies did not recommend neuroimaging and no red flags were reported. Primary headache occurred more frequently in women, in those under 46 years of age with a history of migraine and similar episodes. Moreover, the presence of red flags and the need for neuroimaging in patients with primary headaches were not evidenced.

## Introduction and background

A World Health Organization report on headache disorders draws attention to the global neglect of this important public health problem and reveals the gaps in responses to headaches in countries around the world [1].

Headache is a painful sensation that is in the cranial vault (from the frontal region to the occipital region), including cervical and facial areas [2,3]. More than 50% of outpatient visits for headaches are given in care primary and is a frequent reason for consulting the emergency services where the appropriate approach becomes a fundamental challenge for the clinician from its identification, anamnesis, and correct study, to defining the type of approach to follow [3].

Headache represents between 1%-3% of visits to low and high-complexity emergency departments. Approximately 15% to 20% of patients require neuroimaging of which 0.1% to 5.5% have some relevant pathological finding [4,5]. It mainly affects the active population between 20 and 55 years of age [6], therefore, it is important to study and interpret the red flags based on the SNOOP criteria (systemic symptoms, neurological signs, onset, older age at onset, and prior medical history) [7]; they include the following: age >50 years and other manifestations such as systemic symptoms, neurological signs or symptoms, sudden acute onset, and previous history of headaches to rule out secondary headaches.

Of the headaches studied in the emergency departments, 98% correspond to primary headache, 0.8% to cerebrovascular accidents, 0.6% to intracerebral bleeding, 0.5% to some neuroinfection, and 0.2% to other differential diagnoses such as carotid dissection (a hypertensive disorder associated with pregnancy) and vasculitis [8].

Management of primary headaches, in addition to time constraints, can be challenging in the emergency department setting as a correct diagnostic evaluation is often difficult. The International Classification of Headache Disorders (ICHD) [9] generally refers to the diagnosis of headaches in the outpatient setting.

On the other hand, it is important to highlight that in a study where the ICHD criteria were applied in an emergency services framework, it allowed a correct diagnosis in only two-thirds of the patients [10]. In the same context, an investigation that evaluated the diagnostic accuracy of neurological complaints in the emergency department found that in 35.7% of cases, the initial emergency diagnosis performed by physicians did not coincide with the final diagnosis made later by a neurologist. Thus, primary headaches were found among the most common misdiagnoses [11].

The lack of a clear diagnosis has repercussions on the clinical approach, making it difficult to care for patients in the period after discharge from the emergency department. This aspect reduces the prescription of preventive therapies that generate early readmission to the emergency service in the following days [12]. Furthermore, a recent review on headaches recommends the need to assess prospective studies, which, due to their level of evidence, allow answering questions that help clinicians in the management of these disorders [13].

This is the first systematic scoping review of prospective studies that makes it possible to elucidate the aspects that must be considered in the management of primary headaches in the emergency department.

This systematic scoping review aims to answer the following questions: What are the main characteristics of primary headaches, including age and gender? Is neuroimaging required in patients with a primary headache? Is it possible that some red flags are associated with a primary headache?

## Review

Materials and methods

PRISMA guidelines were followed in this study. Thus, a systematic scoping review of the scientific literature was carried out to perform an analysis of prospective studies that have investigated primary headaches using the Preferred Reporting Items for Systematic Reviews and Meta-Analyses (PRISMA) extension for scoping reviews [14].

The search strategy included the MEDLINE/PubMed, Scopus, LILACS, and SCIELO databases, as well as the grey literature (Google Scholar and Open Grey). MeSH terms and keywords were used to search for prospective articles conducted in all languages (from the oldest record until December 2022) including the terms primary headache, primary headache management, emergency departments, migraine, unspecified headache, ICHD criteria, tension-type headache, cluster headache, or cluster type and trigeminal autonomic headache.

Subsequently, an exploring route was applied to survey databases running Boolean operators (AND, OR): “primary headache management” AND “emergency departments” AND “migraine” AND “unspecified headache” AND “ICHD criteria” AND “tension-type headache” AND “cluster headache” OR “cluster type” OR “trigeminal autonomic headache”.

Research related to drug treatments, pregnant women, case reports, case series, duplicate studies, in vitro experiments, and animal studies were excluded.

Two authors of the present study independently assessed titles and abstracts for eligibility for full-text review. Disagreements were resolved by discussion.

The bibliographic references of relevant reviews and those of selected articles were also manually checked to include studies that had not been included in the electronic or grey literature review. In the selected studies, the authors of the research, the year of publication, the number of people studied with primary headache, their mean age, the proportion of sexes, the main characteristics of the headache, the diagnoses, and triggers of the headache were identified. The need for neuroimaging and the presence or absence of red flags in patients with primary headaches were also presented.

Two authors of this systematic scoping review independently assessed the methodological quality of the included investigations using the Newcastle Ottawa scale that assessed the quality of nonrandomized studies [15]. This scale consisted of 8 items that covered three domains (selection, comparability, and results). Zero to nine stars were assigned, then, the quality of the studies was classified into three groups (low=less than 6 stars; moderate=between 6 and 7 stars; high=8-9 stars).

Results

In the present study, the initial search strategy yielded 670 articles of which 27 corresponded to prospective studies whose full text was reviewed. Finally, only six prospective observational investigations met the selection criteria [5,16-20]; three studies were of moderate methodological quality and three studies of low quality [15].

**Figure 1:**
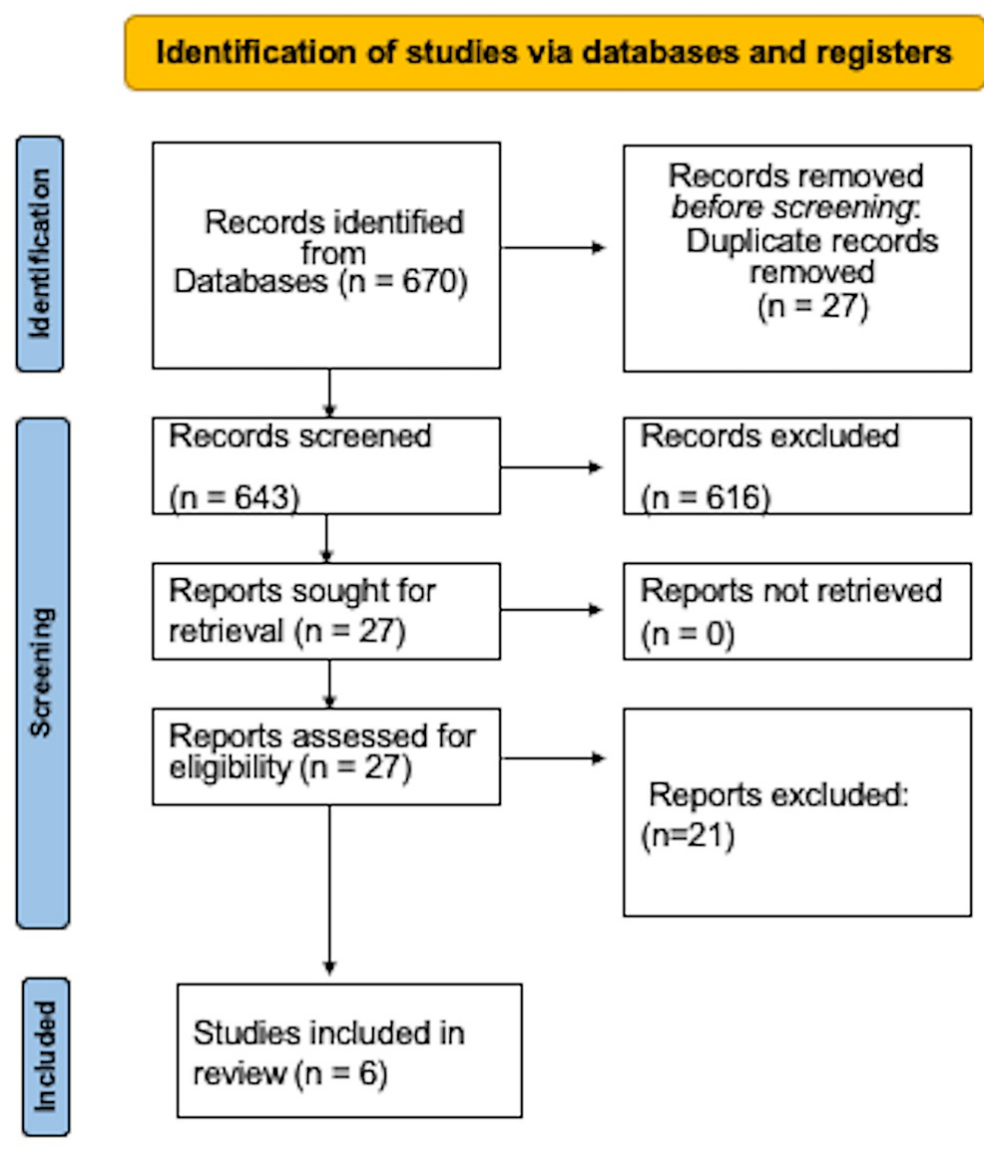
Flowchart of the search strategy and the selection process.

Figure 1 details the flowchart of the search strategy and the selection process.

Table 1 describes the most relevant findings that aim to answer the questions raised in this scoping review. The six selected studies evaluated 600 patients with sample sizes between 14 and 302 participants [16-17] with follow-up periods between seven weeks [5] and 30 months [16]. The included investigations were published between 1989 [16] and 2020 [18].

**Table 1:** Main findings of primary headache in the selected studies. ICHD: International Classification of Headache Disorders; CSF: cerebrospinal fluid.

Authors	Number of patients	Follow-up	Mean age	Proportion women/ men	Main Features	Diagnostics	Triggers	Neuroimaging	Red flags	Quality
Munoz-Ceron et al. [5]	181	7 weeks	39 years	77.8/21.2 %	Absence of aura [54%] Aura presence [25%] Status migrainosus [21%]	ICHD Criterion 3[80.1%] Unspecified primary headache[11.6%] Painful cranial neuropathies and facial pain [8.3%]	Migraine history History of similar episodes Criterion 3B de ICHD	No required	Not reported	Moderate
Harling et al. [16]	14	30 months	Not reported	Not reported	Loss of consciousness[14%] Nausea/vomiting [29%] Neck stiffness[57%] Photophobia [64%] Persistence of pain > 2h [79%]	Migraine [29%] Musculoskeletal headache [29%] Psychogenic headache [36%] Unspecified primary headache [6%].	Exercise, weight lifting, or sexual intercourse	CT, CSF and normal angiography in all patients	Not reported	Low
Locker et al. 17]	302	3 months	Not reported	Not reported	Severe headache[55%] Nausea/vomiting [12%]	Migraine [22%] Unspecified primary headache[18%] Tension headache[11%]	No report	The report is not clear	Not reported	Moderate
Vigano et al. [18]	49	6 months	41 years	82/18%	Aura presence [35%] Persistence of pain > 24h [41%] Nausea/vomiting [35%] Loss of consciousness [6%]	Unspecified primary headache[65.3%] ICHD Criterion 3[34.7%]	Not reported	Not required	Not reported	Moderate
Lledo et al. [19]	15	3 months	43.9 years	47/53 %	Nausea/vomiting [60%]. Severe headache[60%] Severe headache [40%]	Unspecified primary headache [100%]	Migraine history History of similar episodes	Normal CT in all patients	Not reported	Low
Duarte et al. [20]	39	12 months	46.2 years	Not reported	Medium intensity pain [62%] Nausea/vomiting [38%] Aggravated by Valsalva [38%] Wake up from sleep [29%]	Tension headache Migraine	Not reported	Normal CT in all patients	Not reported	Low

Only four studies reported the mean age of people with primary headaches [5,18-20], with ages ranging from 39 [5] to 46 years [20].

Regarding the presence of primary headache according to sex, it was found that of the three studies that reported it, two indicated a higher frequency in women. Only the study by Lledo et al. [19] reported a slightly higher proportion in men.

Within the characteristics of primary headache, most studies reported the presence of nausea/vomiting [16-20], between 12% [17] and 60% [20] of patients. Moreover, the occurrence of intense [17,19] and moderate pain [20], loss of consciousness [16,18], neck stiffness [16], presence of aura [5,18], and photophobia were also described [16].

Regarding the triggers of the primary headache, two studies pointed out a history of migraine and similar episodes [5,19] and one investigation reported exercise, weightlifting, or sexual intercourse [16].

Five studies detailed that the initial diagnosis of primary headache and its follow-up; they were carried out by neurologists or emergency physicians [5,17-20]. The most frequent diagnoses of primary headache included unspecified headache [5,16-19], migraine [16-19], tension-type headache [17,20], and ICHD criterion 3 [5,18].

All the studies indicated that it is not necessary to order neuroimaging [5,16-20]; however, some of them revealed their use [16,19,20], reporting complete normality in their findings. Similarly, none of the studies evaluated showed the presence of red flags in the patients studied.

Three studies were classified as low quality as seen in Table 1. It is important to highlight that the studies included in this systematic scoping review presented great heterogeneity in their designs and great variability in the characteristics of the patients studied, among other characteristics. For these reasons no statistical analysis was performed

As a summary of the main findings presented in Tables 1, we presents some characteristics that clinicians should consider for the appropriate management of primary headaches in emergency departments (Table 2).

**Table 2:** Main findings in the management of primary headaches in emergency departments.

Age	Sex	Feature	Diagnostics	Triggers	Neuroimaging	Red flags
< 46 years	Women	Nausea/vomiting Severe/moderate pain	Unspecified headache Migraine	Migraine history and similar episodes	Not required	None

Discussion

In the emergency departments, the evaluation of the patient with headache must be carried out in a timely and efficient manner due to the concern for possible secondary headaches and the immediate risks that they potentially pose [7]. Bearing in mind that most of the headaches in the emergency departments are primary [8], in this systematic scoping review, some relevant aspects of this type of headache are precisely described; they may allow an adequate and pertinent approach.

Considering that it has been reported that the initial diagnosis of headache made in the emergency department does not coincide with the final diagnosis made later by a neurologist, and seeing that the most common misdiagnosis is related to primary headache [11], this scoping review evaluated prospective studies that could longitudinally assess patients which can generate more reliable results.

The results of this systematic scoping review show that primary headaches occurred more frequently in patients younger than 46 years. In this regard, different studies have shown that patients older than 50 years may have more secondary headaches [21,22]. In this same context, a recent review on the presence of red flags in headaches indicates that the probability that a patient seeking care for a headache decreases with age, while the risk of a serious underlying cause increases 10-fold at age ≥65 years compared with a younger population [13].

In this review, it was found that primary headaches occurred more commonly in women. A meta-analysis [23] and population research [24] that studied the prevalence of headaches according to sex corroborate these results. This can be attributed in part to the effects of estrogen. Estrogen influences Ca2+ and Mg2+ levels, creating an imbalance that can increase neuronal excitation and induce a headache [25].

Regarding the main characteristics of primary headache, most of the studies selected in this review reported the presence of nausea/vomiting [16-20], severe pain [17,19], and moderate pain [20]. There is controversy in the scientific literature about the presence of these findings. Although the most predictive characteristics of the diagnosis of migraine include nausea, vomiting, photo, and phonophobia, it has been indicated that these symptoms together in addition to the intensity of the pain are not useful in the emergency departments to establish a difference. This is due to the high probability of finding these same symptoms in headache syndromes, regardless of their etiology [26]. Moreover, it is important to highlight that there is no classification to score nausea or vomiting associated with the headache, but they are part of the clinical manifestations of some primary headaches.

It has also been reported that, although the intensity of pain due to primary headache is slightly higher in patients treated in emergency departments, compared to those evaluated in ambulatory medical services, it is possible that more than the pain itself, the perception of pain, and the presence of stressful events, and/or psychiatric comorbidities (anxiety or depression), may explain the decision to seek medical care in the emergency department during a headache attack [27].

In this scoping review, it was found that the main triggers of primary headaches include a history of migraine and similar episodes [5,19]. In this regard, some studies have described that at least half of the patients with primary headaches, examined in the emergency services, indicate that these patients have previously experienced similar pain [27-29].

The most frequent diagnoses of primary headache, described in this study, included unspecified headache [5,16-19], migraine [16-19], tension-type headache [17-20], and ICHD criterion 3 [5,18].

It has been described that tension-type headache is the most common type of primary headache [26], while others have indicated that it is a migraine followed by unspecified headache [27]. These diagnostic differences may be due to overcrowding in emergency services, the short time to obtain a detailed clinical history of the headache, and the difficulty in obtaining an accurate history of the patient's headache during the painful phase. Furthermore, the applicability of the ICHD criteria in an emergency setting may be complex [18,30].

The studies evaluated in this review did not indicate neuroimaging during the diagnosis of primary headaches. These results are corroborated by two systematic reviews [31,32], one of them conducted by members of the American College of Emergency Physicians (ACEP) [31]. However, it has been indicated that primary headache frequently leads to unnecessary tests and imaging. For those patients presenting to the emergency department with primary headaches, 2% received lumbar punctures and 14% received neuroimaging [33].

To determine which patients with headaches need additional studies, clinicians and guidelines have traditionally used the presence or absence of red flags. Red flags include fever, neoplasm history, neurologic deficit (including decreased consciousness), sudden or abrupt onset, positional headache, and painful eye with autonomic features, among others [34]. These red flags are related to secondary headaches that occur due to a pathology or condition that predisposes them to appear. This argument has motivated the search for different strategies that may be useful to establish which patients will need more studies, and which patients will need only symptomatic relief, depending on the origin of the headache, and whether it is of primary etiology or not [5].

The investigations selected in this study found no red flags related to a primary headache that would indicate the need for additional measures. However, the same ACEP has pointed out that the applicability of red flags in a busy emergency department has been unfavorable and when applied, did not reduce hospital admissions [35]. It is important to note that red flags should be interpreted with caution to determine the correct neuroimaging indication, bearing in mind that having a red flag does not necessarily mean finding an alteration in the image that leads to a clinical change.

Even though prospective observational studies were selected in this systematic scoping review that would allow reliable conclusions to be drawn, it is necessary to point out that very few investigations with this design have been carried out in the area of headaches; moreover, the investigations included in this study presented great heterogeneity and they were of moderate and low quality. This epidemiological design also increases the risk of selection bias. These studies were very heterogeneous in terms of objectives, patient selection, diagnostic references, analyses, and outcome variables. These shortcomings have also been reported by other reviews about the study of headaches [13,31,35,36].

## Conclusions

Considering the limitations of this systematic scoping review, it is concluded that primary headache occurs more frequently in women, in those under 46 years of age with a history of migraine and similar episodes. Furthermore, the articles selected in this systematic scoping review showed that the presence of red flags and the need for neuroimaging in patients with primary headaches were not evidenced. Medical history and physical examination are essential during the evaluation of a patient with a primary headache because clinical decisions cannot be made without appropriate information. The approach to primary headache includes controlling symptoms and improving functionality with adequate control of the patient's pain.
